# Projected Rapid Habitat Expansion of Tropical Seagrass Species in the Mediterranean Sea as Climate Change Progresses

**DOI:** 10.3389/fpls.2020.555376

**Published:** 2020-11-16

**Authors:** Pedro Beca-Carretero, Mirta Teichberg, Gidon Winters, Gabriele Procaccini, Hauke Reuter

**Affiliations:** ^1^Department of Theoretical Ecology and Modelling, Leibniz Centre for Tropical Marine Research, Bremen, Germany; ^2^Dead Sea-Arava Science Center, Masada, Israel; ^3^Department of Ecology, Leibniz Centre for Tropical Marine Research, Bremen, Germany; ^4^Eilat Campus, Ben-Gurion University of the Negev, Eilat, Israel; ^5^Stazione Zoologica Anton Dohrn, Naples, Italy; ^6^Faculty for Biology and Chemistry, University of Bremen, Bremen, Germany

**Keywords:** biogeographical changes, global warming, *Halophila decipiens*, *Halophila stipulacea*, invasive spread, salinity, species distribution model

## Abstract

During the last 150 years, the tropical seagrass species *Halophila stipulacea* has established itself in the southern and eastern parts of the Mediterranean Sea. More recently (2018), *Halophila decipiens* was observed for the first time in the eastern Mediterranean, and was described as the second non-native seagrass species in the Mediterranean Sea. We implemented a species distribution model (SDM) approach to (1) hindcast the habitat suitability of *H. stipulacea* over the last 100 years in the Mediterranean basin, and (2) to model the increase in the potential habitat suitability of *H. stipulacea* and *H. decipiens* during the current century under two very different climate scenarios, RCP 2.6 (lowest carbon emission scenario) and RCP 8.5 (highest carbon emission scenario). In addition, a principal component analysis (PCA) and *k*-means cluster based on temperature and salinity drivers were applied to visualize the distance and relatedness between the native and invasive *H. stipulacea* and *H. decipiens* populations. Results from this PCA suggest that the *H. stipulacea* populations of the Mediterranean and Red Sea are likely to be similar. In contrast, *H. decipiens* from the Mediterranean is more related to the Atlantic populations rather than to the Red Sea populations. The hindcast model suggests that the expansion of *H. stipulacea* was related to the increases in seawater temperatures in the Mediterranean over the last 100 years. The SDMs predict that more suitable habitat will become available for both tropical species during this century. The habitat suitability for *H. stipulacea* will keep expanding westward and northward as the Mediterranean continues to become saltier and warmer. In comparison, the SDMs built for *H. decipiens* forecast a restricted habitat suitability in the south-eastern Mediterranean Sea at the present environmental conditions and predicts a progressive expansion with a potential increase in habitat suitability along 85% of the Mediterranean coastline. The predicted rapid expansion of non-native seagrass species could alter the Mediterranean’s seagrass community and may entail massive impacts on associated ecosystem functions and services, impacts that have severe socio-economic consequences.

## Introduction

Ocean warming, the increment of frequency and severity of extreme events such as marine heatwaves or storms, increases in salinity, and biological invasions are emerging as severe threats for marine ecosystems, resulting in a significant loss of biodiversity, functionality and associated ecosystem services (i.e., Rilov and Galil, 2009; Gattuso et al., 2015; Oliver et al., 2019). As a consequence, distributional shifts, new biotic interactions, and species extinctions are expected to alter local community compositions (Yang and Rudolf, 2010; Montero-Serra et al., 2015; Kleisner et al., 2017). In coastal ecosystems, seagrasses are key foundation species that create highly productive habitats and nursery grounds for fish and invertebrates, and provide essential functions including CO_2_ sequestration, production of organic carbon and nutrient cycling (Duarte et al., 2010; Costanza et al., 2014; Nordlund et al., 2018). Seagrass habitats, however, are being lost worldwide at unprecedented rates in response to climate change and intensification of human pressures on nearshore coastal ecosystems (Waycott et al., 2009). Loss of seagrass ecosystems involves not only the loss of the associated biodiversity, but also leads to the loss of their associated goods and ecological services (Duarte, 2000; Jordà et al., 2012).

The Mediterranean basin is undergoing tropicalization where its waters are becoming warmer and saltier. Seawater warming rates in this enclosed sea are four times faster than the average of the warming rates of coastal waters of the world (Bianchi and Morri, 2003; Borghini et al., 2014), and these rates are predicted to increase as climate change progresses during this century (Somot et al., 2006; Rilov and Galil, 2009). During the last century, sea surface temperatures (SST) in the Mediterranean Sea have increased at an average rate of +0.04 ± 0.1°C per decade, while in the eastern Mediterranean basin these rates were specially high with summer increases of +0.12 ± 0.07°C year^–1^; surface salinity has increased at an average rate of +0.015 ± 0.002 per decade, and summer increases of +0.008 ± 0.006 year^–1^ in the eastern Mediterranean basin (i.e., Axaopoulos and Sofianos, 2010; Borghini et al., 2014; Ozer et al., 2017).

The opening of the Suez Canal (1869) caused the vast spreading of tropical species from the Red Sea into the Mediterranean (Caspers, 1980). These Lessepsian migrations are expected to increase during this century not only due to the recent doubling of the shipping lane of the Suez Canal which will double the number of ships (Galil et al., 2017), but also, because the current and predicted environmental conditions within the Mediterranean Sea are becoming increasingly more suitable for warm-adapted and high salinity tolerant tropical species (i.e., Bianchi, 2007; Zenetos et al., 2010).

Autochthonous Mediterranean seagrass communities commonly include the large and slow-growing endemic seagrass species *Posidonia oceanica* (L.) Delile and the small and fast-growing species *Cymodocea nodosa* (Ucria) Ascherson, species with different ecological attributes, growth strategies and stress tolerances (e.g., Olesen et al., 2002; Procaccini et al., 2003; Ruiz et al., 2015). *Zostera noltii* and *Ruppia* spp. are also found in more confined areas within the Mediterranean, while *Zostera marina* has a smaller distribution range (Short et al., 2007). The two most abundant species (*P. oceanica* and *C. nodosa*) adopt different strategies in response to temperature increase (Marín-Guirao et al., 2018). *In situ* evidence alongside experimental and modeling studies further confirm that ocean warming will compromise *P. oceanica*’s distribution range as climate change progresses (i.e., Marbà and Duarte, 2010, Marba et al., 2014; Chefaoui et al., 2018; Traboni et al., 2018), while it seems that *C. nodosa* will be resilient to future climate changes in the Mediterranean Sea (Chefaoui et al., 2018; Ontoria et al., 2019).

Native to the Red Sea, Persian Gulf, and Indian Ocean, *Halophila stipulacea* arrived in the Mediterranean Sea shortly after the opening of the Suez Canal (one of the first ever Lessepsian migrants), and for the last 150 years has established itself in most of the eastern and southern Mediterranean Sea (Lipkin, 1975; Gambi et al., 2009). *H. stipulacea* is a small tropical seagrass species that can grow in a wide range of depths (1–70 m), light intensities, salinities (24–70 [partial salinity units]), temperatures (15–34°C), nutrients levels and substrates, making this species an exceptional competitor for resources and space (Steiner and Willette, 2015; Oscar et al., 2018; Beca-Carretero et al., 2020a; Winters et al., 2020). Currently, this species is distributed from the eastern to the central (Sicily coast, Italy) Mediterranean Sea, but it is expected to spread into the west Mediterranean basin as temperature and salinity increase (Gambi et al., 2009; Nguyen et al., 2020). In 2002, *H. stipulacea* was reported in the Caribbean Sea, and during the last 18 years, it has rapidly expanded to most of the Caribbean island nations and has even reached the South American continent (i.e., Vera et al., 2014; Willette et al., 2014; Winters et al., 2020). While its expansion has been so far slow in the Mediterranean Sea (Georgiou et al., 2016), its colonization has been rapid in the Caribbean Sea (i.e., Smulders et al., 2017; reviewed by Winters et al., 2020). Recent studies reported that growth rates from the Caribbean Sea are 8–30 times that of the native Red Sea populations (Azcárate-García et al., 2020; Willette et al., 2020). Recent studies from the Caribbean Sea have additionally shown that *H. stipulacea* is physically displacing local Caribbean species, spreading its dominance within invaded habitats and changing the Caribbean’s seagrass landscape (Willette and Ambrose, 2012; Steiner and Willette, 2015; reviewed by Winters et al., 2020).

A recent study reported the presence of the seagrass *Halophila decipiens* Ostenfeld in Salamina island for the first time (Saronikos Gulf, Aegean Sea, Greece) (38° N), making this species the second non-indigenous seagrass species in the Mediterranean Sea (Gerakaris et al., 2020). *H. decipiens* is considered the only real pan-tropical seagrass species, being broadly distributed in tropical, sub-tropical and warm-temperate nearshore systems in both the southern and northern hemispheres (i.e., Short et al., 2007). *H. decipiens* is generally an annual clonal species with a short life span (6 weeks), high turnover rates and an ephemeral nature present only in warmer months of the year and usually arising from seed banks (Fonseca, 1989; Hammerstrom et al., 2006). Gerakaris et al. (2020) suggested that the recently reported population could be only one year old due to the small size of the patchy meadows. The authors hypothesized that these plants were probably introduced through ballast waters of ships moving from the Red Sea to the Mediterranean Sea. Over the last 5 years, *H. decipiens* has also been expanding its distribution into subtropical Brazilian waters of the southern Atlantic (São Sebastião, 23°44S, 45°20W; Gorman et al., 2016, 2020), suggesting the potential invasive character of this species.

The aims of the present study were to (i) improve our understanding of the trait distance and relatedness between *H. stipulacea* and *H. decipiens* populations from the Mediterranean Sea and other worldwide populations, (ii) assess whether increases in SST over the last 100 years in the Mediterranean Sea could partially explain the colonization of *H. stipulacea* in this basin, and finally, (iii) examine the potential habitat expansion of *H. stipulacea* and *H. decipiens* in the Mediterranean basin during this century by implementing Species Distribution Models (SDMs) in relation to SSTs and salinities. SDMs have been widely applied to investigate the potential biogeographical shifts in response to the effects of global environmental changes of native and invasive terrestrial and aquatic species, including seagrass (i.e., Valle et al., 2014; Chefaoui et al., 2018; Beca-Carretero et al., 2020b; Gamliel et al., 2020). Here, we hypothesize that a wide potential expansions of the habitat suitability of *H. stipulacea* and to a lesser degree of *H. decipiens* are expected to occur during this century as the Mediterranean Sea becomes warmer and saltier. Understanding how invasive seagrass species will respond to different scenarios of global changes is particularly important since changes in the Mediterranean seagrass community will entail impacts on associated ecosystem functionality affecting all neighboring trophic levels and entailing severe socio-economic consequences to neighboring human populations. Despite their critical relevance, modeling studies, incorporating a set of traits to investigate the potential expansion of invasive tropical seagrass species in the Mediterranean Sea due to global change, are limited.

## Materials and Methods

### Seagrass Species Occurrences and Environmental Variables

Data of distribution and presence of *H. stipulacea* and *H. decipiens* were obtained from different sources including published literature and online databases (e.g., the Global Biodiversity Information Facility [GBIF], 2020), detailed along with their date of access and DOI in the Supplementary Material (Supplementary Table S1 and Supplementary Figure S1).

Temperature and salinity have been identified as some of the most important environmental descriptors controlling seagrass growth, survival and distribution; they control physiological processes such as photosynthesis and respiration, but also affect reproduction, flowering and seed germination (i.e., Lee et al., 2007; Oscar et al., 2018; Beca-Carretero et al., 2020a; Nguyen et al., 2020; Winters et al., 2020).

To hindcast the habitat suitability of *H. stipulacea* over the last 100 years (1920, 1950, 1970, and 2019) in the Mediterranean basin, we used SST data derived from the Extended Reconstructed Sea Surface Temperature (ERSST)^1^ (i.e., Meng et al., 2018). This is a monthly global dataset of SSTs, where missing data were filled by applying statistical methods. Collinearity of the environmental variables at a regional scale (presence of *H. stipulacea* in its native areas) were assessed using the Pearson correlation coefficient (*r* > 0.85) (Werneck et al., 2011). We used a final set of 6 environmental and continuous variables of SST including the winter (the months of January–March) and summer (July–September) months. Data were produced with a 2° × 2° resolution (2° is equal to 222 km). The rest of the monthly SST data were excluded due their high collinearity (*r* > 0.85).

To perform the population analysis and the SDMs of both *H. stipulacea* and *H. decipiens*, we used environmental predictors of temperature and salinity available for present (2020) and future climate scenarios (2050 and 2100) of two contrasting greenhouse gas concentration projections including RCP 2.6 (representative concentration pathway; lowest carbon emission) and the RCP 8.5 (highest carbon emission) (IPCC, 2014). Collinearity of the environmental variables at the regional scale (presence of the species) were assessed using the Pearson correlation coefficient (*r* > 0.85). We used a final set of five environmental variables including the mean, maximum and minimum values of SST and maximum and minimum surface salinity, which were continuous variables with a 30 arc-seconds (500 × 500 m) resolution (Tyberghein et al., 2012)^2^. Mean salinity was excluded from the analysis due its high collinearity (*r* > 0.85).

### Population Analysis

To visualize distance and relatedness between the native and invasive populations of *H. stipulacea* and *H. decipiens*, we applied a principal component analysis (PCA) based on a *k*-means cluster analysis for each species (Ding and He, 2004). *K*-means clustering works by assigning a number of centroids based on the number of clusters given. The combination of CA and *k*-means cluster analysis allowed to reduce the dimensions of the dataset and group the different seagrass populations based on the similarity of their environmental conditions (sea surface temperature and surface salinity). Both statistical techniques, PCA and *k*-means cluster, were based on a Euclidean similarity matrix. Two resemblance levels (ellipses) for the PCA and k-means cluster analysis were applied for each seagrass species (*H. stipulacea* and *H. decipiens*). Green ellipses represent levels of 0.035 and red ellipses levels of 0.07. For *H. stipulacea*, we used populations from the Red Sea, Mediterranean Sea, Caribbean Sea, Indian coast, Eastern Africa and Persian Gulf, which correspond with the known populations to date. For *H. decipiens*, we selected populations from the Red Sea, Mediterranean, Arabian Sea, Canary Islands, Bermuda, Caribbean Sea and South American coast, Australian coast, Fiji, French Polynesia, Gulf of California, Andaman Sea, South Chinese Sea and Java Sea (Table 1 and Supplementary Figure S1).

**TABLE 1:** Summary of the environmental descriptors, temperature (°C), and salinity (PSU), of the biogeographical regions of *Halophila stipulacea* and *H. decipiens*.

*Halophila stipulacea*	Min. Temp.	Mean Temp.	Max. Temp.	Min. Salinity	Max. Salinity
Mediterranean Sea	15.2 ± 1.1	21.4 ± 1.1	28.3 ± 1.2	37.3 ± 1.7	39.2 ± 0.5
Red Sea	21.4 ± 1.7	25.3 ± 1.5	29.4 ± 1.3	37.9 ± 0.9	39.6 ± 0.4
Persian Gulf	17.9 ± 0.9	27.2 ± 0.7	34.4 ± 0.3	35.5 ± 1.6	39.2 ± 0.5
Caribbean Sea	25.9 ± 0.4	27.8 ± 0.2	29.8 ± 0.1	32.4 ± 0.8	36.4 ± 0.2
Eastern Africa	24.9 ± 1.1	27.1 ± 0.8	29.5 ± 0.5	32.7 ± 0.9	35.4 ± 0.1
Indian Ocean	26.3 ± 0.3	28.6 ± 0.4	30.9 ± 0.3	29.7 ± 1.3	35.3 ± 0.1
Average values	21.9 ± 4.2	26.2 ± 2.4	30.4 ± 2.0	34.3 ± 2.9	37.5 ± 1.9
*Halophila decipiens*					
Mediterranean Sea	13.9 ± 0.0	20.4 ± 0.0	27.7 ± 0.0	32.4 ± 0.0	38.9 ± 0.0
Red Sea	23.5 ± 2.0	27.6 ± 1.4	31.3 ± 1.2	36.2 ± 0.9	39.1 ± 0.7
Canary Islands	18.3 ± 0.2	21.3 ± 0.2	25.0 ± 0.3	36.4 ± 0.1	37.1 ± 0.1
Arabic Sea	23.9 ± 0.3	26.5 ± 0.5	29.8 ± 0.5	35.4 ± 0.1	36.9 ± 0.1
Caribbean Sea	22.1 ± 2.1	26.8 ± 0.7	30.4 ± 0.5	34.9 ± 1.3	36.5 ± 0.3
South American Coast	23.9 ± 0.3	26.1 ± 0.5	28.6 ± 0.5	35.2 ± 0.1	37.0 ± 0.1
Bermuda Islands	18.6 ± 0.0	23.3 ± 0.0	28.5 ± 0.0	36.1 ± 0.0	36.8 ± 0.0
Gulf of California	18.2 ± 0.0	24.8 ± 0.0	30.3 ± 0.0	33.7 ± 0.0	35.2 ± 0.0
French Polynesia	25.8 ± 0.0	27.7 ± 0.0	29.3 ± 0.0	35.3 ± 0.0	36.6 ± 0.0
Andaman Sea	27.8 ± 0.0	29.4 ± 0.0	31.3 ± 0.0	30.3 ± 0.0	33.6 ± 0.0
South Chinese Sea	23.4 ± 0.0	27.3 ± 0.0	30.1 ± 0.0	29.9 ± 0.0	34.3 ± 0.0
Java Sea	27.4 ± 0.0	28.9 ± 0.0	30.5 ± 0.0	30.6 ± 0.0	34.5 ± 0.0
Fiji	24.7 ± 0.01	27.0 ± 0.0	29.5 ± 0.0	33.8 ± 0.0	35.7 ± 0.0
Coral Sea	21.1 ± 1.5	25.4 ± 1.1	29.1 ± 0.7	34.0 ± 0.7	35.9 ± 0.2
Tasmanian Sea	15.3 ± 1.6	19.6 ± 1.6	23.4 ± 0.9	35.3 ± 0.1	35.7 ± 0.1
Southwest Australia	22.6 ± 1.1	27.4 ± 1.1	31.2 ± 0.3	31.6 ± 1.0	35.6 ± 0.1
Northwest Australia	16.8 ± 0.0	19.6 ± 0.0	22.5 ± 0.0	35.2 ± 0.0	35.9 ± 0.0
Average values	21.6 ± 4.1	25.1 ± 3.2	28.6 ± 2.6	33.8 ± 2.1	36.2 ± 1.4

*Max, maximum; Min, minimum. Data are presented as means ± SD.*

### Species Distribution Models (SDMs)

To determine potential habitat suitability of the two seagrass species in the Mediterranean Sea, we used the MAXENT model, which is based on a maximum-entropy algorithm and has a consistent predictive performance using only occurrence records (Elith and Leathwick, 2009). As there is a potentially wider distribution of *H. stipulacea* and *H. decipiens* in the Mediterranean Sea because these species may be continually expanding into new areas, and also because there are no specific monitoring program that follow-up the spreading of either species in this basin, we consider that models using only occurrence records are more appropriate than models using presence and absence records (Franklin, 2010). MAXENT (Phillips et al., 2006) was run with the default response settings. Firstly, a SDM was preformed to investigate the potential effect of SST rise over the last 100 years (1920, 1950, 1970, and 2019) in the Mediterranean Sea on the habitat expansion of *H. stipulacea* in this basin. To hindcast the habitat suitability of *H. stipulacea* we only used data of the target species in its native area, excluding the records from the Mediterranean Sea (Supplementary Figure S1). Secondly, additional SDMs were applied to (i) investigate the potential variation in their biogeographical range experienced during this century, (ii) to determine the main environmental drivers determining the habitat preferences of the target species, and (iii) to assess the annual linear coastline potentially suitable as new habitats for colonization. The model was built with the following assumptions: (i) the distribution of the seagrass (*H. stipulacea* and *H. decipiens*) has remained constant since first records of its distribution, (ii) the presence of the target species is in equilibrium with the current environmental descriptors, and (iii) there is no exclusion through competition with other species. MAXENT generated a continuous raster file with a pixel value ranging from 0 to 1, with 0 representing the absence of the target species, and 1 representing the highest probability for potential habitat suitability. The predicted probabilities derived from the SDMs were transformed into classified maps with two possible categories: suitable habitat or unsuitable habitat. To assume the potential habitat suitability of the seagrass, we used two different logistic thresholds. To hindcast the habitat suitability of *H. stipulacea* over the last 100 years (1920–2019) based on SST, we used the “10 percentile training presence” threshold which corresponded to 0.13. To model the present and future habitat expansion of *H. stipulacea* and *H. decipiens* we used the logistic threshold “equal training sensitivity and specificity” which corresponded to 0.152 for *H. stipulacea* and 0.278 for *H. decipiens* (Supplementary Table S2).

**FIGURE 1:**
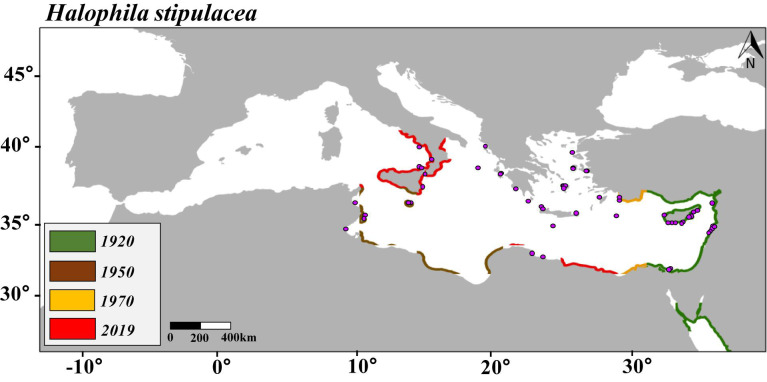
Predicted maps of habitat suitability and distribution of *Halophila stipulacea* over the last 100 years (1920, 1950, 1970, and 2019) based on sea surface temperature (SST). Purple points represent the current distribution of *H. stipulacea* in the Mediterranean Sea.

**FIGURE 2:**
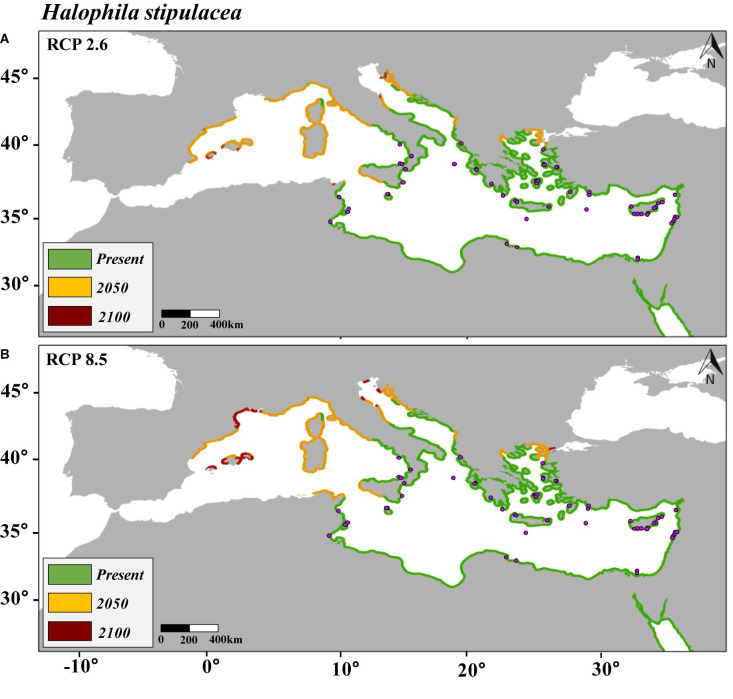
Maps of the distribution of predicted habitat (salinity and temperature) suitability for *Halophila stipulacea* in the Mediterranean Sea under present conditions and under future scenarios of climate change of two contrasting carbon emission projections (RCP 2.6 **[Panel A]** and RCP 8.5 **[Panel B]**) by 2050 and 2100. Note that yellow is additional habitat to the present and red additional to 2050. Purple points represent the current distribution of *H. stipulacea* in the Mediterranean Sea.

**FIGURE 3:**
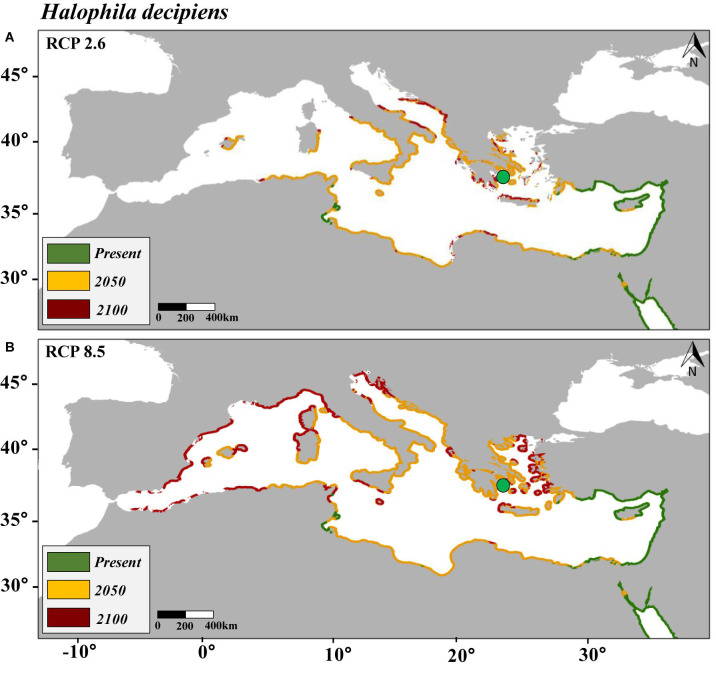
Maps of the distribution of predicted habitat (salinity and temperature) suitability for *Halophila decipiens* in the Mediterranean Sea under present conditions and under future scenarios of climate change of two contrasting carbon emission projections (RCP 2.6 **[Panel A]** and RCP 8.5 **[Panel B]**) by 2050 and 2100. Note that yellow is additional habitat to the present and red additional to 2050. Purple points represent the current distribution of *H. decipiens* in the Mediterranean Sea.

Data of the species records were divided five times into a calibration group (80%) and a validation group (20%). Final maps of distribution (Figures 1–3) were obtained based on the average of the five independent predictions. The SDMs were evaluated using two different evaluation measures chosen according to the nature of the data and the SDMs. The first evaluation approach was based on the threshold-independent metric “area under the curve (AUC)” “receiver operating characteristic (ROC)”; ROC values between 0.5 and 0.7 indicated that the model is performing poorly; values from 0.7 to 0.9 were considered to have a moderate discriminatory ability, and models with values higher than 0.9 were considered to perform excellently (Manel et al., 2001). The significance of the AUC was tested using a cross-validation procedure covering 100 interactions. The second evaluation approach was the sensitivity parameter, which is the proportion of the presence of the target species correctly predicted by the SDMs (Allouche et al., 2008).

Finally, we assessed the potential annual linear coastline that could become potentially suitable as new habitat for colonization based on SST and surface salinity (km year^–1^). The annual linear coastline was calculated as the number of new kilometers of coastline which are suitable to be colonized by *H. stipulacea* and/or *H. decipiens*, excluding those kilometers where the SDMs built for the present scenario predicts the distribution of the species. The modeled Mediterranean basin accounts for a total of 36,600 km of coastline.

## Results

This study encompassed an updated review of the distribution of *H. stipulacea* and *H. decipiens* in the studied area (Supplementary Table S1 and Supplementary Figure S1). The SDM built to hindcast the habitat suitability of *H. stipulacea* over the last 100 years (1920–2019) (Figure 1) had an AUC of 0.97 and a sensitivity of 92% (Supplementary Table S2). The SDMs built to model the present and future habitat suitability of *H. stipulacea* and *H. decipiens* (Figures 2, 3) showed a marked discriminatory ability, with AUC values of 0.94–0.96, and a high sensitivity, with MAXENT models accurately predicting 94 and 96% of the records of *H. stipulacea* and *H. decipiens*, respectively (Supplementary Table S2). Results of the relative contribution of the environmental descriptors indicated that the most important variables of the SDMs built for *H. stipulacea* were maximum temperature with a contribution of 70.2% and maximum salinity (18.4%) (Table 2). While the most relevant environmental descriptors for *H. decipiens* were minimum and maximum temperatures with a contribution of 31.9 and 29.9%, respectively, followed by maximum salinity (25.2%).

**TABLE 2:** Scores (%) of the relative contributions of the environmental variables (sea surface temperature and salinity) based on their permutation importance to the Maxent models built for *Halophila stipulacea* and *H. decipiens*.

Relative contribution (%)	*H. stipulacea*	*H. decipiens*
Minimum temperature	4.4	31.9
Mean temperature	1.6	10.4
Maximum temperature	70.2	29.9
Minimum salinity	5.4	2.6
Maximum salinity	18.4	25.2

The SDM built to hindcast the habitat suitability of *H. stipulacea* over the last 100 years (1920–2019) showed a progressive habitat expansion as the Mediterranean Sea became warmer. Compared with the 2,947.6 km of the Mediterranean’s coastline that was predicted by the SDM to be a suitable habitat for *H. stipulacea* in 1920, our SDMs predicted 3,924.2 km in 1950, 4,467.9 km in 1970, and 7,686.6 km in 2019 (Supplementary Figure S2). By 2019, the SDM built for *H. stipulacea* adequately predicted the presence of 62% of the Mediterranean records that occur today (Figure 1).

**FIGURE 4:**
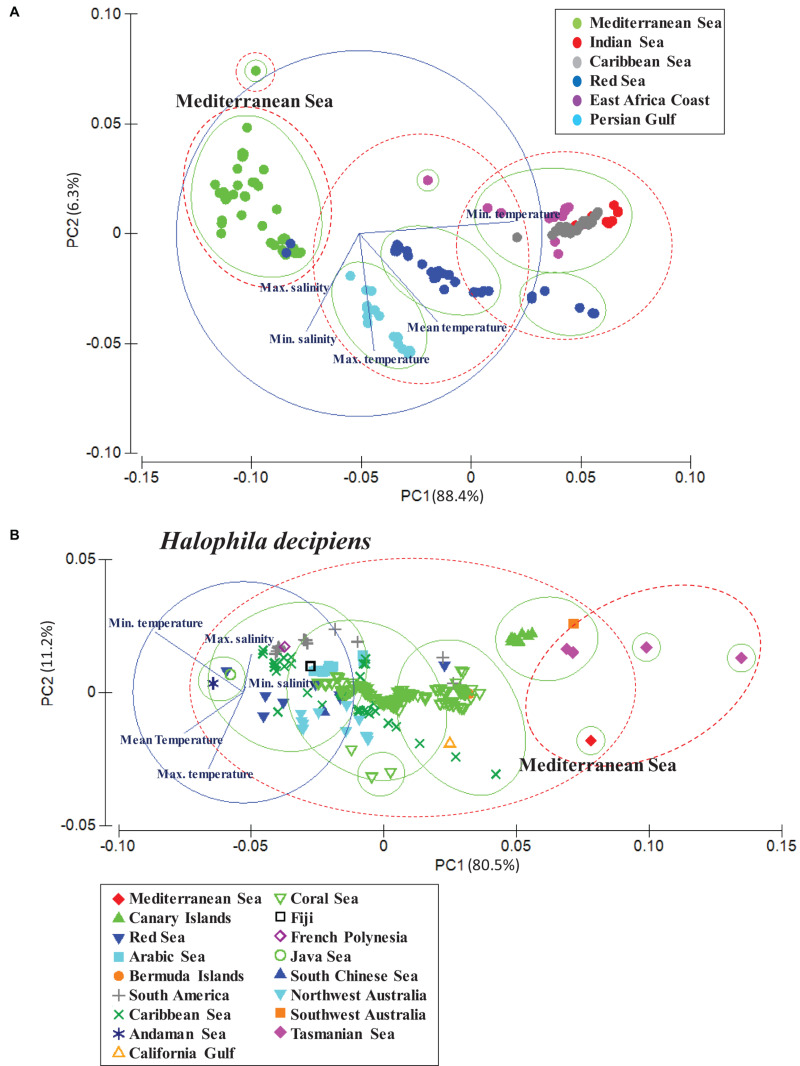
Results of PCA based on the cluster analysis (Supplementary Figure S3) revealing the relatedness and dissimilarity of *Halophila stipulacea* **(A)** and *H. decipiens* populations **(B)** in relation to the mean, maximum and minimum values of sea surface temperature (SST) and maximum and minimum of surface salinity (Tyberghein et al., 2012, www.bio-oracle.org).

The PCA and *k*-means cluster grouped Mediterranean and some of the Red Sea *H. stipulacea* records in the same group, indicating that these populations are exposed to similar environmental conditions (Figure 4A). Compared with Red Sea *H. stipulacea* populations, the Mediterranean Sea *H. stipulacea* populations were distributed in significantly colder average temperatures (*t*-test, *p* < 0.05), while there were no significant differences of maximum temperatures (Mediterranean Sea = 29.4 ± 1.3°C; Red Sea = 28.3 ± 1.2°C) (Table 1). Mean, minimum and maximum salinities of the Mediterranean and Red Sea populations did not show significant differences. In addition, PCA clustered native *H. stipulacea* populations from the Indian Sea and East Africa coast in the same group with the invasive Caribbean Sea population. In contrast, the PCA of the *H. decipiens* populations reported that the environmental conditions from the Mediterranean Sea (only one record) were more related to warm-temperate adapted populations, such as populations from Tasmania Sea, southwest Australia or Canary Islands populations than to the tropical Red Sea populations (Figure 4B). For instance, the Mediterranean (20.4 ± 0.0°C) and Canary Island (21.3 ± 0.2°C) populations were distributed in significantly (*t*-test, *p* < 0.05) colder habitats than the Red Sea populations (27.6 ± 1.4°C). Similar trends were found for salinity values, whereby the Mediterranean and Canary Islands plants were distributed in significantly (*t*-test, *p* < 0.05) less salty waters than the Red Sea populations (Table 1).

**FIGURE 5:**
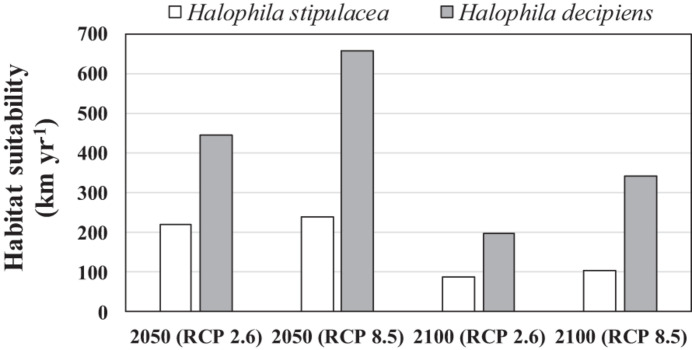
Average length of coastline (km year^− 1^) of suitable new habitat (compatible in terms of salinity and temperature) predicted by the species distribution models built for *Halophila stipulacea* and *H. decipiens* over two periods (2020–2050 and 2050–2100) under future scenarios of climate change of two contrasting carbon emission projections (RCP 2.6 and RCP 8.5).

The current westernmost distribution of *H. stipulacea* in the Mediterranean Sea was observed in the Palinuro harbour (Italy, 40.03°N, 15.27 E°) (Gambi et al., 2009, 2018; Di Genio et al., 2020) in the central part of the Mediterranean basin, and the coast of Sousse (Tunisia, 35,9° N, 10.6 E) (Sghaier et al., 2014) in the south (Supplementary Figure S1). However, the SDM built for *H. stipulacea* for the present climate scenario predicted a habitat suitability in further western areas, such as the Island of Corsica (France, 42.7° N, 8.5° E) (Figure 2A). The SDMs built for *H. stipulacea* under future climate projections showed a marked range expansion into northern and western areas as seawater temperatures and salinity levels increase in the Mediterranean Sea. By 2050, the SDMs predicted an average annual increase of the habitat suitability of 221 km year^–1^ under the scenario RCP 2.6 and 239 km year^–1^ under the scenario RCP 8.5, although these habitat increase rates significantly decreased after 2050 (87 km year^–1^) (Figure 5). By 2100, the SDM predicted a continued westward expansion reaching areas of western Spain (40.5° N, 1.0° E) and the Balearic Islands. By 2100 under the worst scenario of carbon emission (RCP 8.5), the SDM predicted a habitat suitability of 30,271 km, which represents 83% of the total Mediterranean modeled coastline (Table 3 and Figure 2B).

**TABLE 3:** Kilometers of coastline of suitable sea surface temperature and salinity habitat predicted by the species distribution models (SDMs) built for *Halophila stipulacea* and *H. decipiens* under present and future scenarios (2050 and 2100) of climate change of two contrasting carbon emission projections (RCP 2.6 and RCP 8.5).

		2050		2100	
	Present	RCP 2.5	RCP 8.2	RCP 2.5	RCP 8.2
*H. stipulacea*	21,795	28,436	28,956	28,752	30,271
*H. decipiens*	3,594	17,007	23,299	19,459	31,003

*The Mediterranean basin account for a total of 36.600 km of coastline.*

In comparison, the SDM built for *H. decipiens* for the current climate scenario predicted a habitat suitability of 13,413 km of coastline which represents t 37% of the entire Mediterranean coast (Table 3). The fundamental niche (defined as the range of environments where a species is found) of *H. decipiens* in the Mediterranean Sea is divided into two main areas: (i) the south-eastern coast including Cyprus (34.7° N, 33.1° E), and (ii) to a lesser degree, the Strait of Sicily (Tunisia; 33.85° N, 10.37° E) (Figure 3). By 2050, under the RCP 2.6 and 8.5 scenarios, the SDMs predicted similar projections; however, by 2100 the SDMs predicted two contrasting scenarios. Between the present distribution and 2050, the models predicted an average increase in habitat suitability of 323 km year^–1^ under both RCP 2.6 and 8.5, primarily connecting areas of habitat suitability predicted by the present climate scenario and expanding into the central and western part of the Mediterranean basin, reaching the Balearic Islands (40.17° N, 3.88° E) (Figure 5). In contrast, between 2050 and 2100, the SDMs showed two different predictions for RCP 2.6 and 8.5 carbon emission scenarios. Under the RCP 2.6 scenario, the SDM built for *H. decipiens* predicted a habitat suitability of 19,459 km with a suitable habitat expansion of 198 km year^–1^, potentially covering 53% of the Mediterranean coastline. Habitat suitability under the scenario of higher carbon emissions of RCP 8.5 is predicted to expand westward and northward into the Mediterranean basin potentially reaching 31,002.7 km of coastline, representing 85% of the basin, with a habitat expansion of 342 km year^–1^ (Table 3 and Figure 5). Interestingly, for both *H. stipulacea* and *H. decipiens* the SDMs of 2050 and 2100 were not predicting the loss of suitable habitat distribution under both scenarios of carbon emission RCP 2.6 and RCP 8.5.

## Discussion

Future climate projections estimate that marine Mediterranean ecosystems will experience the most substantial worldwide change in biodiversity (i.e., Rilov and Galil, 2009; Huntley, 2017; Corrales et al., 2018). Predicted increasing temperatures of ∼3–4°C and salinity levels of ∼1.0–1.2 PSU by the end of this century in the Mediterranean Sea (IPCC, 2014) could exceed the adaptive capacity of some native species and cause the migration or disappearance of their ecological niche. This will result in local distribution losses and the emergence of new habitats potentially available for colonization by better-adapted species (Jordà et al., 2012). Climate change is expected to compromise the distribution of the large and slow-growing endemic Mediterranean seagrass species *P. oceanica* and, to a lesser degree, the fast-growing *C. nodosa* (i.e., Olesen et al., 2002; Chefaoui et al., 2018; Ruocco et al., 2018; Ontoria et al., 2019). In contrast, increases in temperature and salinity are expected to promote the performance and distribution of the invasive species *H. stipulacea* (Oscar et al., 2018; Nguyen et al., 2020; Wesselmann et al., 2020). Our results clearly show a critical expansion of the biogeographical range of *H. stipulacea* and *H. decipiens* as climate change progresses during this century, with 80% of the Mediterranean coastline predicted to be suitable for colonizing based on temperature and salinity conditions alone. However, there is less evidence of the invasive capacity of *H. decipiens*, as this species was just recently found for the first time in 2018 along the coast of Greece (38°N) (Gerakaris et al., 2020).

Our hindcast model was implemented using only SST, while our SDMs were implemented with only one additional environmental parameter (SST and surface salinity) (i.e., Chefaoui et al., 2018; Wilson et al., 2019; Gamliel et al., 2020). Temperature and salinity are some of the main factors defining the physiological thresholds and distribution of seagrasses (Lee et al., 2007; Marbà and Duarte, 2010), although, other factors (biochemical, physical and biological) may also contribute to determining seagrass habitat ranges. A well-known limitation of SDMs applied to past and future assessments in marine ecosystems is the scarcity of accessible environmental descriptors over large temporal and spatial scales, as well as depicting the multitude of interactions intrinsic for complex natural systems. On local scales biological interactions such as grazing or competition, physical settings (light availability, wave exposure) or chemical water composition (most importantly nutrient concentration) are decisive if a species can settle and if a population can survive and persist. Therefore, our study should be taken as a proxy for the potential change of the fundamental niche of *H. stipulacea* and *H. decipiens* in relation to SST and salinity. Our SDMs were built on a regional scale; therefore, fine-scale factors were not incorporated into these models, such as biological interactions or dispersion mechanisms alongside some environmental factors including current velocity, wave exposure or sediment type. These, could potentially limit or even enhance the presence and expansion of *H. stipulacea* and *H. decipiens* into new predicted habitats (Greve and Binzer, 2004; Kraan et al., 2020). Previous modeling studies reported that the most important variables defining the distribution of seagrass species for present climate conditions at a local scale were salinity, irradiance, depth and nutrient concentrations (Fourqurean et al., 2003; Kraan et al., 2020). Conclusively, with future large-scale availability of other parameters which have been proven to affect seagrass performance such as pH, irradiance or nutrients (i.e., Lee et al., 2007; Winters et al., 2020) the model complexity will increase and will result in a more accurate spatial prediction of suitable habitats.

While *H. stipulacea* in the Mediterranean Sea is considered a Lessepsian migrant, it is native to the Red Sea, Persian Gulf, and the Indian Ocean (Winters et al., 2020). Results from the PCA revealed that the environmental conditions, at least at the surface, from the Mediterranean populations are actually more similar to Red Sea conditions than to Indian or Persian Gulf waters. Interestingly, PCA results also showed that environmental conditions from the Caribbean *H. stipulacea* populations match more closely to Eastern Africa and Indian Ocean populations than to the Mediterranean populations. Over the last 150 years, the expansion of *H. stipulacea* in the Mediterranean has been related with two main factors, the opening of the Suez canal which allowed the entrance of native species from the Red Sea to the Mediterranean, and the increase of seawater temperatures experienced over the last decades in this region (reviewed by Winters et al., 2020).

Our SDM built to hindcast the colonization of *H. stipulacea* in the Mediterranean basin over the last 100 years suggested that the habitat expansion of *H. stipulacea* was correlated with the recorded increases in SST. The hindcast model predicted the presence of *H. stipulacea* in 62% of the current records in the Mediterranean Sea, while the remaining 38% of the points were not predicted. This may suggest that other environmental factors which are not incorporated in the model may more adequately explain the expansion of *H. stipulacea* into northern latitudes during the last century. Other factor that could constrain the habitat suitability of the target species in northern regions of the Mediterranean Sea is the fact that we only used records of *H. stipulacea* from its native regions to perform the hindcast model. Nevertheless, the model was conducted at a regional scale, and variations of temperature at a local scale due to the local topography, presence of currents or up-welling events may constrain or expand the habitat suitability range of the seagrasses. Yet, the colonization of *H. stipulacea* in the Mediterranean basin has been moderate within an expansion rate of 12 km year^–1^ (Georgiou et al., 2016). Based on the increases in SSTs and surface salinities predicted in the region (discussed above), our SDMs predicted that more suitable coastline habitats will become available to be colonized by *H. stipulacea* with a potential habitat suitability expansion of 239 km year^–1^ by 2050 under the worst carbon emission scenario (RCP 8.5). By the end of 2050, in both scenarios of carbon emission (RCP 2.6 and 8.5), the habitat suitability of *H. stipulacea* is predicted to reach the Iberian Peninsula and the Balearic Islands in the westernmost coast of the Mediterranean basin. The SDMs pointed out that maximum annual temperatures, and to a lesser degree maximum annual salinity, are essential factors determining the fundamental niche of *H. stipulacea* in the Mediterranean Sea. Our results are consistent with recent experimental studies demonstrating the first evidence of optimal acclimatization of native and invasive Mediterranean populations of *H. stipulacea* to projected scenarios of Mediterranean climate change, including temperatures of 30–32°C and salinities higher than 40 PSU (Georgiou et al., 2016; Oscar et al., 2018; Nguyen et al., 2020; Wesselmann et al., 2020). Similarly, native populations from the Persian Gulf are adapted to even more extreme conditions, with *in situ* summer temperatures of 33–34°C and summer salinities of 39.0–39.5 PSU (Tyberghein et al., 2012), defining *H. stipulacea* as an exceptional euryhaline and thermal-tolerant species. Adaptability to such high salinities and temperatures suggests the potential capacity of this species to successfully thrive and compete for space and resources under future climate conditions in the Mediterranean Sea.

From the present scenario to 2100, the increase of suitable areas available to be colonized by *H. stipulacea* is predicted to be moderate, with an annual increase of 97 km year^–1^. Although this rate is considered slow in comparison with other marine macrophyte invaders (∼300–400 km year^–1^), such as the macroalgae *Caulerpa* and *Sargassum* spp. (i.e., Lyons and Scheibling, 2009; Mineur et al., 2015), it will provide the opportunity for this species to expand into 83% of the Mediterranean coastal area, with only the north of the Adriatic Sea and the southwest of the basin as environmentally unsuitable areas for its distribution. This habitat restriction is potentially explained by the relatively cold winter conditions and upwelling events that maintain SST lower than 13–14°C, which might represent a physiological minimal threshold for the survival of *H. stipulacea* (Gambi et al., 2009). While the presence of cold waters may represent a barrier to the expansion of *H. stipulacea* into westernmost habitats in the Mediterranean Sea, a recent study identified this region as a habitat refuge for the foundation species *P. oceanica* and *C. nodosa* under future climate scenarios (Chefaoui et al., 2018). Similarly, an even newer study reported that *H. stipulacea* in the Mediterranean Sea can cope with significantly lower temperatures (∼14°C) than what observed in its native habitats (∼23°C), suggesting a thermal niche shift to the lower temperatures in the Mediterranean Sea and potentially allowing this species to colonize temperate regions (Nguyen et al., 2020; Wesselmann et al., 2020). This physiological capability may be explained by the capacity to produce and accumulate high levels of unsaturation in the lipidic structures of the leaves of *H. stipulacea* (Beca-Carretero et al., 2019). In the northern Red Sea, *H. stipulacea*’s lipid composition, which partially determines the thermal tolerance of primary producers (Murakami et al., 2000), is characterized by high levels of n-3 polyunsaturated fatty acids (PUFAs), more related to species from the Mediterranean Sea (*P. oceanica* and *C. nodosa*) and the temperate species *Z. noltii* (Portugal, 37°N) than to tropical species. Finally, SDMs did not predict any loss in the *H. stipulacea* habitat suitability range under any climate scenario, indicating a potential stable habitat distribution in the Mediterranean basin.

Until recently, the spread of *H. stipulacea* in the Mediterranean basin was considered to be from clonal propagation as these plants exclusively developed male flowers (Procaccini et al., 1999; Gambi et al., 2009). However, the recent documentation of both female and male flowers (Nguyen et al., 2018) and mature seed capsules in the Mediterranean (Gerakaris and Tsiamis, 2015) demonstrates its capacity for sexual reproduction in this invasive habitat, suggesting a potential increase in its genetic diversity. Genotypic diversity, the production of seed banks and seed recruitment have been documented to have positive effects on seagrass production, enhancing resilience and recovery to different stresses, including storms, diseases, or warming events (i.e., Ehlers et al., 2008; Reynolds et al., 2012; Rasheed et al., 2014). The recent enlargement of the Suez Canal (July 2015; Galil et al., 2017) may also favor the arrival of new populations of *H. stipulacea*, further contributing to the interaction between different genotypes. Another factor that can favor the expansion of *H. stipulacea* in the Mediterranean basin is its proved capacity to acclimate and survive in disturbed areas under high nutrient concentrations and human pressures, such as harbors or polluted bays in both native (Red Sea; Beca-Carretero et al., 2020a) and non-native regions (Mediterranean and Caribbean Sea) (Gambi et al., 2009; Willette and Ambrose, 2012; Van Tussenbroek et al., 2016; Beca-Carretero et al., 2020a). However, in an ongoing study we observed that *H. stipulacea* populations from Eastern Africa were highly vulnerable to experimental warming and nutrient increase conditions, suggesting different populations-specific stress-tolerance and resilience capacity (Viana et al. in revision).

While Gerakaris et al. (2020) suggested that *H. decipiens* was probably introduced from the Red Sea to the Mediterranean, our PCA analysis indicated that the environmental conditions of the Mediterranean were more related to the conditions of warm-temperate waters from the south hemisphere or North Atlantic, such as, Canary Islands, Bermuda or Caribbean Sea, and indicated that these populations were more likely to be closely related than that of the Red Sea population. Nevertheless, a genetic study will be necessary to confirm the origin of the Mediterranean population. Our models indicated that *H. decipiens* could become a future invader in the Mediterranean Sea and potentially establish itself in 40–80% of the shallow waters of this basin during this century; however, some factors can compromise its further colonization and stabilization. The habitat expansion of *H. decipiens* was previously reported in warm-temperate Brazilian waters of the southern Atlantic, where this species has been expanding and firmly developing extended monospecific meadows as ocean warming progresses (Gorman et al., 2016, 2020). Similarly, in the Canary Islands, a consistent expansion of *H. decipiens*’ distribution range has been shown since 1980 (Pavón-Salas et al., 2000; Ruiz et al., 2015); this was suggested to potentially be associated with the reported increase in SSTs at a rate of 0.2°C/decade since 1970 (Luque et al., 2013).

In the Mediterranean Sea, *H. decipiens* was only reported in Salamina Island (Greece), however for the present climate scenario, it was predicted to grow in one-third of this basin. Our SDMs suggested that based on the environmental conditions required for this species, it could already inhabit other Mediterranean regions. However, the fact that this species was not reported in more Mediterranean coastal areas could indicate that some factors may restrict its entrance and spread in the basin. For instance, the low abundance of *H. decipiens* populations in the Red Sea may restrict its successful colonization into the Mediterranean Sea. Furthermore, two main processes have been related with the success of species invasions: death and reproduction (Williamson and Fitter, 1996; Sakai et al., 2001). In its native habitat, this species can adapt to a wide range of temperatures, including anomalous winter temperatures reaching 13–14°C in Bermuda in the Atlantic Sea (23.3°N, −64.8°O) (Manuel et al., 2013). These observations suggest that Mediterranean winter temperatures will not represent a critical physiological threshold for this species, thus not preventing its survival from year to year. In its native habitat, *H. decipiens* colonizes new areas by propagating via seed transport or vegetative fragments (Hammerstrom et al., 2006). Gerakaris et al. (2020) observed flowers in an early stage of maturity in Greece, suggesting potential expansion via sexual reproduction and seed production, although this hypothesis has not been confirmed. However, if the species does not produce seeds, its settlement and dispersal into the Mediterranean basin may be limited. Remarkably, its recently reported occurrence provides a unique case study to examine whether non-native seagrass species can acclimate in a new habitat and evolve within their new environmental conditions.

The coexistence of native Mediterranean seagrass species (e.g., *P. oceanica*, *C. nodosa*, and *Z. noltii*) with invasive species (such as *H. stipulacea* and *H. decipiens*) enables potential biological interactions between them, and the possibility of generating diverse response mechanisms, such as competition, exclusion, or species facilitation. Indeed, similar questions arise with *H. stipulacea* and *H. decipiens* as the SDMs predicted large areas where their fundamental niche can overlap. To date, both species were observed forming mixed assemblages in the Caribbean Sea (Willette et al., 2014; Winters et al., 2020); however, there is no clear evidence of the consequence of their potential interactions over time. Recent reports from the Mediterranean Sea (eastern Tunisian coast) have demonstrated first signs of species interactions showing a potential physical replacement or displacement of the local seagrass *C. nodosa* by the invasive *H. stipulacea* (Sghaier et al., 2014). Studies from the Caribbean have shown that *H. stipulacea* is physically displacing local Caribbean species (Willette and Ambrose, 2012; reviewed by Winters et al., 2020). However, to date, there are no mechanistic explanations of competition between invasive and native seagrass species. In the context of climate change, the adaptability of *H. stipulacea* and *H. decipiens* to predicted Mediterranean conditions suggests their potential to successfully thrive and compete for space and resources with native Mediterranean Sea seagrass species. In this basin, studies predicted a potential loss of habitat suitability of *P. oceanica* as climate change advances during this century in the Mediterranean Sea (Marbà and Duarte, 2010; Marba et al., 2014), and to a lesser extent of *C. nodosa* (Chefaoui et al., 2018). The increase in frequency of extreme weather events such as heatwaves, storms and “hurricanes” (Cavicchia et al., 2014) and other local factors such as nutrient pollution, which have been found to be detrimental to native seagrass populations (Danovaro, 2003) in the Mediterranean Sea, will lead to more frequent disturbance of seagrass habitats. Although *P. oceanica* has been shown to be much more resilient to temperature increases than previously thought and may have escape mechanisms such as flowering (Marín-Guirao et al., 2018, 2019), its loss by human-related impacts may provide an opportunity for fast-growing and rapid colonizing seagrass species to spread into these unoccupied areas. Moreover, both *H. stipulacea* and *H decipiens* are some of the deepest-adapted seagrass species worldwide, often occurring at more than 30 m but also growing at a depth of 60–70 m, (i.e., Duarte, 1991; Coles et al., 2009; Winters et al., 2020). This exceptional capacity may allow these invaders to occupy the potential habitat loss of deep-adapted Mediterranean seagrass meadows which were reported to be highly vulnerable to anomalous increments in water temperature (Marbà and Duarte, 2010; Marba et al., 2014). Therefore, as a consequence of climate change, we may predict a possible transition from Mediterranean seagrass communities with relatively large seagrass species, diverse ecological attributes and growth strategies, to a community dominated by small and fast-growing species. The potential re-structuring of the native Mediterranean seagrass communities may lead to impacts on associated goods, ecological services and ecosystem functionality affecting all the trophic levels (i.e., Fourqurean et al., 2012; Jordà et al., 2012; Viana et al., 2019).

With Mediterranean marine ecosystems facing unprecedented anthropogenic threats, it is critical to understand the potential role that invasive species may play in the configuration of new benthic communities. To better monitor the potential spread of invasive seagrass species in the Mediterranean sea and understand their ecological role, we suggest to (i) implement SDMs to develop habitat conservation and invasive species management plans, including environmental risk assessments to potentially anticipate the arrival of invasive species and effectively implement contingency actions, (ii) create specific areas to continually monitor the vegetative development of invasive species and their potential interaction with native species, and finally (iii) increase public awareness of the impacts of invasive species on native ecosystems and gain support from local divers, fisherman and coastal communities.

## Data Availability Statement

The raw data supporting the conclusions of this article will be made available by the authors, without undue reservation.

## Author Contributions

MT, GW, GP, and HR developed the theoretical idea of the manuscript and collaborated in the final version of the manuscript. PB-C developed the models and statistics and wrote the first draft of the manuscript. All authors contributed to the article and approved the submitted version.

## Conflict of Interest

The authors declare that the research was conducted in the absence of any commercial or financial relationships that could be construed as a potential conflict of interest. The reviewer JCC declared a past co-authorship with one of the authors PB-C.

## Footnotes

1 https://www.ncdc.noaa.gov/

2 www.bio-oracle.org
