# Supplementary material for: Projected Rapid Habitat Expansion of Tropical Seagrass Species in the Mediterranean Sea as Climate Change Progresses
Source: Front Plant Sci. 2020 Nov 16;11:555376. doi: 10.3389/fpls.2020.555376 (PMC7701102; doi:10.3389/fpls.2020.555376)
Supplement: Supplementary file 1 [file Data_Sheet_1.docx]

**SUPPORTING INFORMATION**

**Table S1.** Distribution of *H. stipulacea* and *H. decipiens based on the literature survey.*

| **SPECIES** | **Source of data, date of access, DOI** | **Locations** |
| --- | --- | --- |
| *Halophila stipulacea* | GBIF (<http://www.gbif.org/>; accessed 5^th^ Dec 2019) https://doi.org/10.15468/dl.aubofk | Red Sea, Mediterranean Sea, Caribbean Sea, |
|  |  | Indian Ocean and Eastern Africa. |
| *Halophila stipulacea* | Winters et al. 2020 | Red Sea, Mediterranean Sea, Caribbean Sea, |
|  |  | Indian Ocean and Eastern Africa |
| *Halophila stipulacea* | Wesselmann et al. 2020 | Red Sea and Mediterranean Sea |
| *Halophila stipulacea* | Anton et al. 2020 | Red Sea |
| *Halophila decipiens* | GBIF (<http://www.gbif.org/>; accessed 5^th^ Dec 2019) https://doi.org/10.15468/dl.wmiycc | Canary Islands, Caribbean Sea, South |
|  |  | American coast, French Polynesia, Gulf of California, Andaman Sea, South Chinese Sea, Java Sea, Australian populations and Fiji. |
| *Halophila decipiens* | Manuel et al., 2013 | Caribbean Sea |
| *Halophila decipiens* | Gorman et al., 2016 | South American coast |
| *Halophila decipiens* | Ruiz et al. 2015 | Canary Islands |
| *Halophila decipiens* | Gerakaris et al. 2020 | Mediterranean Sea |
| *Halophila decipiens* | Jacobs & Dicks, 1985 | Red Sea |
| *Halophila decipiens* | Qurban et al. 2019 | Red Sea |
| *Halophila decipiens* | Anton et al. 2020 | Red Sea |
| *Halophila decipiens* | Den Hartog, 1989 | Arabic Sea |
| *Halophila decipiens* | Schils & Coppejans, 2003 | Arabic Sea |
| *Halophila decipiens* | Milchakova et al., 2005 | Arabic Sea |
| *Halophila decipiens* | Ibrahim, 2018 | Arabic Sea |

**Figure S2.** Current distribution of *Halophila stipulacea* and *H. decipiens* based on the literature survey and online databases listed in table S1**.**

**Figure S3.** Kilometres of coastline of suitable habitat based on sea surface temperature (SST) predicted by the species distribution models (SDMs) built to hindcast the potential expansion of *H. stipulacea* over the last 100 years (1920, 1950, 1970 and 2019) in the Mediterranean basin. The Mediterranean basin account for a total of 36.600 km of coastline.

**Figure S4.** Dendrogram showing mutual similarities of *Halophila stipulacea* (Panel A) and *H. decipiens* (Panel B) populations worldwide. Solid lines show the different groups formed by the CLUSTER analyses.

**Table S5.** Characteristics of the MAXENT models built for *Halophila stipulacea* and *H. decipiens* including the area under the receiver operating characteristic curve (AUC) and sensitivity, and the logistic threshold.

| **Model Characteristics** | | ***H. stipulacea (1920-2019)*** | ***H. stipulacea***  ***(present -2100)*** | ***H. decipiens***  ***(present -2100)*** |
| --- | --- | --- | --- | --- |
| Regularized training gain | | 2.270 | 2.415 | 1.695 |
| Unregularized training gain | | 3.189 | 2.783 | 2.141 |
| Training AUC | | 0.97 | 0.98 | 0.952 |
| Sensitivity | | 91.6 | 94.2 | 95.5 |
| Logistic threshold | | 0.13 | 0.2783 | 0.152 |
| Number of interactions | | 500 | 500 | 500 |
| Records used for training | | 18 | 223 | 267 |
| Points used for the SDM | | 10,018 | 10,223 | 10,267 |
| Output format | | Logistic | Logistic | Logistic |
| Regularization multiplier | | 1 | 1 | 1 |
| Max. number of backgrounds points | | 10,000 | 10,000 | 10,000 |
| Replicate run type | | Crossvalidate | Crossvalidate | Crossvalidate |
| Maximum interactions | | 500 | 500 | 500 |
| Convergence threshold | | 0.00001 | 0.00001 | 0.00001 |
| Adjust sample radius | | 0 | 0 | 0 |
| Log file | | maxent.log | maxent.log | maxent.log |
| Default prevalence |  | 0.5 | 0.5 | 0.5 |
